# Cloud–Edge Collaborative Hardware Sensing with Dynamic Reliability Estimation and Evidence-Constrained Large Language Model Reasoning

**DOI:** 10.3390/s26196000

**Published:** 2026-09-22

**Authors:** Liyang Liu, Ziyi Ren, Runxi Chen, Yan Li, Shuwen Yin, Jiahong Pu, Manzhou Li

**Affiliations:** 1 Peking University, Beijing 100871, China; 2 China Agricultural University, Beijing 100083, China

**Keywords:** cloud–edge–end collaboration, hardware anomaly detection, multi-sensor temporal modeling, sensor reliability estimation, device topology modeling

## Abstract

Banking data centers generate heterogeneous IT operational streams from servers, network devices, edge nodes, and business terminals. Existing detectors are vulnerable to asynchronous sampling and unreliable channels, while separate root-cause and language-model modules cannot preserve an end-to-end evidence trail. This study presents a cloud–edge–end framework that couples sensor-quality-aware temporal encoding, topology- and chronology-constrained event-graph reasoning, and evidence-constrained language generation. At the edge, dynamic reliability scores suppress missing, noisy, delayed, drifting, and frozen channels. At the cloud layer, the model ranks root causes and identifies propagation relations and onset times from device topology, logs, alarms, and maintenance records. The language model then generates traceable incident interpretations from the resulting evidence package and rejects claims when support is insufficient or conflicting. Experiments use IT operational data collected from the headquarters and disaster-recovery data centers of a commercial bank between 2024 and 2025; the dataset contains no customer financial or transaction records. The proposed method achieves 95.38% Accuracy, 95.06% Precision, 94.71% Recall, a 94.88% F1-score, and a 97.41% AUC for hardware anomaly detection. Root-cause localization accuracy, Top-3 Accuracy, event-relation F1-score, and temporal localization error are 91.84%, 97.26%, 90.73%, and 1.82 s, respectively. The complete model attains a 94.68% evidence support rate and a 4.26% unsupported statement rate. These results show that reliability is propagated from sensing through system-level diagnosis to evidence-grounded interpretation.

## 1. Introduction

Modern cloud–edge–end infrastructures support safety-critical services and expose device state through processor, power, thermal, storage, environmental, and network sensors [1,2]. Their operational value depends on more than detecting an anomalous window. Operators must determine which device initiated an incident, how the disturbance propagated, when it began, and which observations support the conclusion.

The key problem is that these requirements are coupled but existing methods address them separately. Threshold and conventional machine-learning detectors assume stable, trustworthy inputs and are sensitive to missing observations, clock offsets, drift, and value freezing [3,4,5,6]. Temporal encoders improve sequence modeling but generally concatenate channels or assign fixed fusion weights, so a corrupted channel can dominate the decision [7,8]. Graph models capture topology but usually receive anomaly scores without retaining the quality of the source measurements. Finally, prompt-only or retrieval-augmented large language models can produce fluent diagnoses without enforcing token-level support, weakening auditability [9,10]. The resulting gap is an end-to-end trust problem: uncertainty detected at a sensor must remain visible during root-cause ranking and explanation generation.

This study addresses that gap through a hardware-sensing framework in which reliability is propagated across three coupled stages. First, a quality-supervised estimator converts completeness, stability, delay, and cross-channel agreement into dynamic sensor weights. Second, these weights accompany anomaly events into a topology- and chronology-constrained graph that jointly predicts root nodes, propagation edges, and onset times. Third, graph confidence and source reliability become evidence gates, vocabulary constraints, and rejection signals for language-model decoding. Thus, the technical contribution is not a loose assembly of temporal, graph, and language modules; it is the explicit transfer of calibrated reliability from raw observations to event relations and finally to generated claims.

The framework targets IT operational data in banking data centers. It does not analyze customer financial or transaction data. The application setting is important because service continuity, conservative attribution, and evidence traceability are required during high-load periods such as settlement and disaster-recovery switching. The main contributions are as follows.

1. A reliability-propagation architecture links sensor-level quality estimation, device-level temporal detection, system-level event-graph reasoning, and claim-level evidence control within one trainable workflow.
2. A quality-supervised sensor estimator learns from recorded and physics-consistent corruption states and provides bounded, interpretable weights for missing, noisy, delayed, drifting, and frozen channels.
3. A relation-aware event graph couples topology, chronology, logs, and reliability-weighted sensor events to jointly rank root causes, predict propagation edges, and localize anomaly onset.
4. An evidence-constrained decoder combines source-dependent gates, a claim-bearing vocabulary mask, conflict measurement, and calibrated rejection so that every factual claim remains traceable to a device, time, and evidence source.
5. A multimodal IT-operations benchmark from a commercial bank evaluates anomaly detection, root-cause localization, propagation relations, temporal localization, and explanation support under a leakage-safe chronological split.

## 2. Related Work

### 2.1. Privacy Protection and Secure Reasoning in Cloud–Edge–End Collaboration

Privacy protection and secure reasoning in cloud–edge–end collaborative environments have mainly been developed through four paradigms: differential privacy, homomorphic encryption, secure multiparty computation, and federated learning [11]. Differential privacy reduces sensitive information leakage through noise injection, while homomorphic encryption enables secure inference over encrypted data [12]. Secure multiparty computation and federated learning further support collaborative model training and inference without directly exposing raw data, providing important foundations for distributed intelligent perception and privacy-preserving collaboration [13]. Despite these advances, existing methods remain insufficient for real-time hardware sensing applications [14]. Differential privacy has primarily been studied for static features or text data and rarely considers continuous temporal dependencies or anomaly propagation. Homomorphic encryption still suffers from high computational and communication overhead, limiting its scalability to large models and long sensing sequences [15]. Although federated learning reduces centralized data collection, model updates may still leak sensitive information, and periodic parameter aggregation has difficulty satisfying the requirements of low-latency anomaly detection, dynamic cloud–edge collaboration, and cross-device event reasoning [16]. Overall, current privacy-preserving approaches mainly focus on training protection, encrypted inference, or distributed parameter collaboration, while unified support for real-time heterogeneous sensing, dynamic sensor reliability estimation, cross-device anomaly propagation analysis, and evidence-grounded interpretation remains limited. Furthermore, cloud-centric solutions often increase communication latency, whereas edge-only models lack sufficient capability for complex event reasoning and semantic interpretation, highlighting the need for more integrated cloud–edge–end security reasoning frameworks.

### 2.2. Reliability and Evidence Modeling in Multi-Source Learning

Evidential deep learning represents class probabilities with a Dirichlet distribution and interprets network outputs as evidence, providing an explicit estimate of predictive uncertainty [17]. Trusted multi-view classification extends this principle by aggregating view-specific opinions. Hierarchical opinion aggregation separates intra-view denoising from inter-view fusion [18], while RSEA-MVGNN uses subjective-logic uncertainty to control structural enhancement and multi-view graph aggregation [19]. Recent trusted long-tailed learning further combines opinion aggregation with uncertainty-guided pseudo-data generation [20]. These methods establish that uncertain sources should contribute less to a fused decision.

The present problem differs in both the unit of reliability and its downstream use. A “view” here is a physical sensor channel whose quality changes within an incident window because of missingness, delay, drift, freezing, or cross-sensor inconsistency. The proposed estimator is therefore supervised by channel-quality states rather than only by classification uncertainty. Its score is propagated into event-node confidence, propagation-edge evidence, and language-model gates. This preserves source quality across temporal detection, graph reasoning, and claim generation instead of terminating reliability modeling at the classification output.

### 2.3. Domain Large Models for Trustworthy Intelligent Analysis

Domain large models have recently demonstrated remarkable capabilities in complex semantic understanding, risk analysis, and decision support across domains such as law, contract intelligence, and finance. Representative studies have established standardized benchmarks for legal reasoning [21], enabled automated contract analysis [22], and developed domain-specific financial large language models for intelligent financial analysis [23]. These advances demonstrate the strong potential of domain large models for complex information understanding and structured reasoning [24]. Despite these achievements, existing approaches are still predominantly designed for centralized plaintext data processing [25]. Privacy-preserving reasoning, data minimization, and secure collaboration are rarely incorporated into model architectures [26]. Moreover, current domain large models provide limited support for heterogeneous multi-source sensing data, cloud–edge–end collaborative environments, and evidence-grounded reasoning, making it difficult to generate trustworthy and traceable analytical results in distributed intelligent systems [27]. Overall, although domain large models provide a strong foundation for intelligent analysis, their integration with privacy protection, distributed collaboration, and evidence-based reasoning remains insufficient, motivating the development of a trustworthy cloud–edge–end collaborative security reasoning framework.

### 2.4. Security Incident Impact Assessment and Traceable Analysis

Security incident impact assessment has been extensively studied from the perspectives of economic loss, risk quantification, and compliance analysis [28]. Representative studies estimate the financial consequences of cybersecurity incidents, optimize security investment through economic and game-theoretic models, and employ artificial intelligence to support regulatory compliance assessment [29]. These approaches provide important foundations for evaluating incident consequences and supporting security decision-making [30]. However, existing methods are primarily based on post-incident statistical analysis or analytical optimization models and rarely incorporate real-time system observations [31]. Consequently, they cannot effectively exploit hardware sensing data, device topology, anomaly propagation information, or operational evidence to identify fault origins, reconstruct propagation paths, and assess impact scopes [32]. Moreover, impact assessment and intelligent security reasoning are generally performed independently, limiting the establishment of unified and evidence-grounded decision-making processes [33]. Overall, although current studies provide valuable models for economic assessment, risk analysis, and compliance evaluation, they remain insufficient for real-time, hardware-aware, and traceable security reasoning, motivating the development of integrated frameworks that combine impact assessment with intelligent security analysis.

## 3. Materials and Methods

### 3.1. Data Collection

The dataset comprises IT operational data from server clusters, database nodes, edge gateways, core switches, firewalls, business terminals, and environmental monitoring systems in the headquarters and same-city disaster-recovery data centers of a commercial bank. It contains device telemetry, infrastructure events, logs, maintenance records, topology, and public regulatory text; it contains no customer balances, transactions, account identifiers, or other business-content data. Collection spans January 2024 to December 2025 and covers routine operating hours, overnight batch processing, month-end settlement, system upgrades, maintenance, and peak-load periods, as summarized in Table 1. Redfish/IPMI, operating-system monitors, and GPU telemetry interfaces provide server states. SNMP, NetFlow/IPFIX, and streaming telemetry provide network throughput, latency, packet loss, queue length, and interface state. Modbus/TCP environmental monitors provide cabinet temperature, humidity, and power-supply state. A common clock aligns these sources within each analysis window.

Linux Journald, Windows Event Log, database audit logs, network-device Syslog, and application logs feed a centralized logging platform. Hardware, network, and security platforms provide alarms, while the IT service management system, configuration management database (CMDB), audit platform, and bastion-host records provide maintenance and operation events. CMDB relationships, LLDP/CDP adjacency, traffic dependencies, and service calls define the device topology. Automated alarms, fault tickets, post-incident reports, and two independent annotators determine anomaly labels, onset times, root-cause devices, propagation nodes, and impact scopes. Public laws, regulations, standards, cases, and penalty decisions support only evidence-constrained auxiliary interpretation and do not generate supervised hardware-anomaly labels.

### 3.2. Data Preprocessing and Augmentation

Sensors use different sampling rates, timestamp resolutions, and protocols. Their streams therefore contain misalignment, missing observations, abrupt fluctuations, drift, and heterogeneous scales. Logs, alarms, tickets, topology, and public documents also have discrete, textual, or graph representations. The preprocessing pipeline treats continuous sensing, discrete events, relations, and documents separately. It then applies physics-consistent temporal augmentation to training windows to improve robustness to missingness, noise, time shifts, and compound faults. Let the observation of the *i*-th sensor at time *t* be denoted by $x_{i}\left(t\right)$. The multivariate time series composed of *d* sensor channels is represented as

(1) $$X=\left\{x_{1},x_{2},\ldots ,x_{T}\right\},\;x_{t}\in R^{d},$$

where *T* denotes the number of time steps and *d* denotes the number of sensor channels. Temporal synchronization was performed with respect to a unified reference timeline

(2) $$T=\left\{t_{1},t_{2},\ldots ,t_{T}\right\}.$$

Let the difference between the standard reference time and the local time of the *i*-th device be defined as $\Delta t_{i}=t_{\mathrm{ref}}-t_{i}$. The corrected timestamp is then given by

(3) $$t_{i}^{\prime }=t_{i}+\Delta t_{i}.$$

Clock offsets were jointly determined from Network Time Protocol records, server baseboard management controller timestamps, and centralized logging platform timestamps. After clock correction, all channels were resampled at a unified interval Δτ. For continuously varying variables, including temperature, power consumption, current, voltage, fan speed, network latency, and port traffic, linear interpolation was applied when $t_{a}<t<t_{b}$:

(4) $${\hat{x}}_{i}\left(t\right)=x_{i}\left(t_{a}\right)+\frac{t-t_{a}}{t_{b}-t_{a}}\left[x_{i}\left(t_{b}\right)-x_{i}\left(t_{a}\right)\right].$$

For discrete variables, including device alarms, port states, service states, and task execution states, nearest-neighbor matching or forward filling was adopted to prevent the generation of nonexistent intermediate states. For sensor streams sampled at frequencies higher than that of the unified timeline, aggregation was performed within the resampling window $W_{t}$:

(5) $${\bar{x}}_{i}\left(t\right)=\frac{1}{|W_{t}|}\sum_{\tau \in W_{t}}x_{i}\left(\tau \right).$$

In addition to the mean, the maximum, minimum, standard deviation, and rate of change within each window were retained to characterize instantaneous peaks and local fluctuations. For example, temperature peaks, power fluctuation amplitudes, and maximum packet-loss rates provide complementary evidence for overheating, sudden workload increases, and network congestion. Missing observations were mainly caused by sensor failures, network interruptions, acquisition-agent exceptions, device restarts, and interface invocation failures. Different processing strategies were selected according to the duration of missingness and signal type. Short and approximately random missing segments in continuous variables were imputed using linear interpolation or local medians. Forward filling was applied to discrete state variables, although the filling range was restricted to a predefined temporal window. Long consecutive missing segments were not forcibly interpolated. Instead, their missing states were preserved and transferred to the subsequent reliability estimation module. The missing-value mask of the *i*-th sensor at time *t* was defined as

(6) $$m_{i}\left(t\right)=\left\{\begin{array}{cc} 1, & x_{i}\left(t\right)\;\mathrm{is}\;\mathrm{validly}\;\mathrm{observed},\\ 0, & x_{i}\left(t\right)\;\mathrm{is}\;\mathrm{missing}. \end{array}\right..$$

The elapsed time since the most recent valid observation of the corresponding channel was also recorded:

(7) $$\delta_{i}\left(t\right)=t-\max\left\{\tau <t|m_{i}\left(\tau \right)=1\right\}.$$

The imputed values, missing-value masks $m_{i}\left(t\right)$, and elapsed-time variables $\delta_{i}\left(t\right)$ were jointly fed into the model. Consequently, genuine observations, short-term estimates, and long-term unupdated states could be distinguished, preventing imputed values from being treated as equally reliable as original measurements. Outlier detection was performed to identify abnormal observations caused by transient sensor errors, communication interference, interface parsing errors, or acquisition-program failures. For channels with approximately stable distributions, the standard score was used to quantify the deviation of an observation from the statistical distribution estimated from the training set:

(8) $$z_{i}\left(t\right)=\frac{x_{i}\left(t\right)-\mu_{i}}{\sigma_{i}},$$

where $\mu_{i}$ and $\sigma_{i}$ denote the mean and standard deviation of the *i*-th sensor in the training set, respectively. When $|z_{i}\left(t\right)|$ exceeded the threshold γ, the observation was marked as a candidate outlier. Because hardware sensing data may exhibit skewed distributions, the interquartile range criterion was additionally applied:

(9) $${\mathrm{IQR}}_{i}=Q_{3,i}-Q_{1,i},$$

(10) $$x_{i}\left(t\right)\notin \left[Q_{1,i}-\lambda {\mathrm{IQR}}_{i},Q_{3,i}+\lambda {\mathrm{IQR}}_{i}\right],$$

where $Q_{1,i}$ and $Q_{3,i}$ denote the first and third quartiles, respectively, and λ denotes the outlier detection coefficient. Candidate outliers were not directly removed. When an extreme variation was supported by device alarms, correlated sensor changes, or maintenance records, the observation was retained as a genuine fault signal. When an observation exceeded the physical operating range of the device and was inconsistent with neighboring time points, associated sensors, and system logs, it was classified as an acquisition error and repaired through local interpolation or physical boundary clipping. This strategy prevented genuine temperature surges, power peaks, and network latency increases from being incorrectly eliminated as noise. Because temperature, power consumption, voltage, latency, traffic, and task-queue variables differ substantially in numerical range, standardization based on training-set statistics was applied to eliminate dimensional effects:

(11) $${\tilde{x}}_{i}\left(t\right)=\frac{x_{i}\left(t\right)-\mu_{i}}{\sigma_{i}+\varepsilon },$$

where ε is a small constant introduced to prevent division by zero. The same $\mu_{i}$ and $\sigma_{i}$ estimated from the training set were used for the validation and test sets to avoid information leakage. For variables with pronounced long-tailed distributions, including port traffic, task-queue length, and disk read/write volume, a logarithmic transformation was first applied to compress extreme values:

(12) $$x_{i}^{\log}\left(t\right)=\log\left(1+x_{i}\left(t\right)\right),$$

after which standardization was performed. System logs, device alarms, maintenance tickets, and operation records were first aligned with hardware sensing windows according to unified device identifiers and corrected timestamps. Structured fields, including event type, severity level, target device, service name, operator identity, and execution result, were extracted from Linux Journald, Windows Event Log, database audit logs, network-device Syslog, and application service logs through log-template parsing. Duplicate logs, invalid heartbeat records, and continuously repeated alarms were merged, while device addresses, user identifiers, and business-related fields were anonymized. Log text was subsequently cleaned, tokenized, and semantically encoded to generate textual representations corresponding to device-level anomaly events. Maintenance tickets, configuration changes, and bastion-host operation records were structured according to occurrence time, involved device, operation type, and processing result, and were associated with sensing windows before and after each anomaly.

Device topology and service dependencies were jointly reconstructed from CMDB asset relationships, LLDP/CDP adjacency information, network traffic dependencies, and service call chains. Let the device relationship graph be denoted by G=(V,E), where V represents entity nodes, including servers, database nodes, switches, edge gateways, firewalls, and business terminals, and E represents physical connections, network communications, service invocations, and task dependencies. Connection relationships duplicated across different data sources were merged according to device identifiers and temporal ranges. When conflicting topology information was observed, the final relationship was determined according to the joint consistency among CMDB records, real-time adjacency information, and observed traffic evidence. For laws, regulations, regulatory documents, judicial cases, and administrative penalty decisions, webpage tags, duplicate content, headers, footers, and invalid formatting characters were removed. The documents were then segmented according to legal provisions, case facts, responsible entities, behavioral obligations, violation facts, evidentiary bases, and disposition outcomes. Sensitive identifiers associated with individuals, institutions, and cases were anonymized. The cleaned legal text was transformed into semantic vectors and indexed in a retrievable evidence repository. These data were used exclusively for responsibility-oriented auxiliary interpretation by the cloud-side evidence-constrained large language model and were not involved in generating supervised labels for hardware anomaly categories, root-cause devices, or propagation relationships. After basic preprocessing, continuous time series were segmented using sliding windows with length *L* and stride *S*. The *n*-th sample window was represented as

(13) $$X_{n}=\left[x_{nS},x_{nS+1},\ldots ,x_{nS+L-1}\right].$$

Each window retained standardized observations, missing-value masks, elapsed sampling intervals, local statistical features, log events, and device relationship information, which were organized according to device identifiers as model inputs. The training, validation, and test sets were divided chronologically. The same anomaly event and its adjacent windows were restricted to a single subset to prevent information leakage caused by overlapping windows. Table 2 reports the record-level allocation before windowing. The nine continuous sensor streams contain 7,442,400 records. The chronological 70%/15%/15% split assigns 5,209,680 records to training and 1,116,360 records each to validation and testing. Logs, alarms, tickets, and operation records contain a further 248,420 discrete events, split into 173,894, 37,263, and 37,263 events, respectively. Topology relationships and public documents are indexed as side information and are not augmented.

Augmentation is applied online only to training windows. Each original training window produces one augmented view with probability $p_{\mathrm{aug}}=0.30$ in an epoch; therefore, the augmentation percentage is 30%. At record-equivalent level, expected training exposure increases from 5,209,680 to 5,209,680×1.30=6,772,584 continuous records per epoch, while validation and test sizes remain unchanged. Because augmented views are regenerated online, this value describes expected exposure rather than permanently stored duplicate files. To improve adaptability to complex operating environments, random missingness, noise injection, local masking, temporal shifting, sensor drift, and value freezing were applied only to the training set. Random missingness was introduced through a Bernoulli mask to simulate packet loss and intermittent acquisition failures:

(14) $$x_{i}^{\mathrm{miss}}\left(t\right)=b_{i}\left(t\right)x_{i}\left(t\right),\;b_{i}\left(t\right)∼\mathrm{Bernoulli}\left(1-p_{m}\right),$$

where $p_{m}$ denotes the random missing probability. Noise injection was applied to simulate sensor measurement errors and electromagnetic interference:

(15) $$x_{i}^{\mathrm{noise}}\left(t\right)=x_{i}\left(t\right)+\epsilon_{i}\left(t\right),\;\epsilon_{i}\left(t\right)∼N\left(0,\sigma_{n}^{2}\right),$$

where $\sigma_{n}$ controls the noise intensity. Local masking concealed one or more sensor channels within a randomly selected interval $\left[t_{s},t_{e}\right]$ to simulate local communication interruptions or acquisition-agent failures. Temporal shifting translated a specified channel by $\delta_{i}$ sampling steps along the temporal axis to simulate device clock drift and transmission delay:

(16) $$x_{i}^{\mathrm{shift}}\left(t\right)=x_{i}\left(t-\delta_{i}\right).$$

Sensor drift was introduced to simulate device aging, persistent bias, and calibration errors:

(17) $$x_{i}^{\mathrm{drift}}\left(t\right)=x_{i}\left(t\right)+\alpha_{i}\left(t-t_{0}\right)I\left(t\geq t_{0}\right),$$

where $\alpha_{i}$ denotes the drift rate and $t_{0}$ denotes the drift onset time. Value freezing was introduced to simulate a sensor that ceased updating while continuously returning an outdated observation:

(18) $$x_{i}^{\mathrm{freeze}}\left(t\right)=x_{i}\left(t_{f}\right),\;t\geq t_{f},$$

where $t_{f}$ denotes the freezing onset time. Multiple augmentation operations were combined probabilistically to simulate complex faults, including simultaneous packet loss and sensor drift, sequential fan-speed anomalies and temperature increases, and the co-occurrence of network congestion, task-queue accumulation, and service response delay. However, compound augmentation was not performed in a fully random manner. Constraints were imposed according to device operating mechanisms, anomaly compatibility, and event chronology. For example, a reduction in fan speed could only be combined with a simultaneous or subsequent temperature increase, while packet loss could be associated with subsequent task-queue accumulation and service response delay. Samples with contradictory causal ordering or physically implausible relationships were not generated. The augmented time series was uniformly expressed as

(19) $$X^{\prime }=A_{K}\circ A_{K-1}\circ \cdots \circ A_{1}\left(X\right),$$

where $A_{k}$ denotes the *k*-th augmentation operation and ∘ denotes sequential composition of different operations. The original data distributions of the validation and test sets were preserved, and no random augmentation was applied. Through the above preprocessing and augmentation procedures, hardware sensing sequences, log records, device topology, and legal evidence were converted into model inputs with a unified temporal reference and explicit structural relationships. Consequently, genuine anomaly dynamics and propagation patterns were preserved, while the influence of missing data, noise, drift, sensor freezing, and temporal misalignment was reduced, thereby improving the generalization capability of the proposed method in complex cloud–edge–end computing environments.

### 3.3. Proposed Method

#### 3.3.1. Overall

A cloud–edge–end collaborative security perception framework is constructed by integrating trusted multi-sensor temporal encoding, cross-device anomaly event chain modeling, and evidence-constrained large language model reasoning. The synchronized and normalized multi-channel hardware sensing sequences are first fed into the edge-side temporal encoding module according to device identity and temporal windows. Local variation patterns, including rapid temperature increases, power fluctuations, fan speed degradation, network latency growth, and task queue accumulation, are extracted from each sensor channel using temporal convolutional networks. Subsequently, long-range temporal dependencies and correlations among heterogeneous sensors are modeled through Transformer-based feature interaction. Meanwhile, dynamic reliability scores are estimated according to data completeness, noise intensity, sampling delay, and cross-sensor consistency, enabling reliable channels to receive higher weights while suppressing the influence of missing, drifting, frozen, or abnormal sensor measurements.

Figure 1 summarizes the prediction path. Sensor reliability is estimated at the edge and attached to every device-level event. The event graph combines this confidence with topology and time to score candidate roots and propagation edges. The retained nodes, edges, sensor segments, and text records form an evidence package whose reliability is consumed by the decoder gate, mask, conflict estimator, and rejection branch. The final output is therefore either a device- and time-specific supported explanation or an explicit uncertainty statement. The reliability-weighted multi-channel representations are aggregated into device-level state embeddings, which are further processed by anomaly classification and temporal localization branches to generate anomaly categories, occurrence probabilities, starting timestamps, critical sensor channels, and their contribution scores. These outputs are transformed into standardized device-level anomaly events. Subsequently, anomaly events generated from different devices are transferred into the cross-device event chain construction module, where structured event graphs are constructed by jointly incorporating device topology, service invocation relationships, system logs, and operational records. In the constructed graph, nodes represent abnormal states of specific devices within corresponding time windows, while edges represent physical connections, network dependencies, task interactions, temporal orders, or potential propagation relationships.

Through aggregation of neighboring device states and event relationships, the graph model estimates the probability of each abnormal node being the root cause and predicts whether direct propagation relationships exist among events. Consequently, the initial abnormal source, direct causes, affected devices, and propagation paths are obtained. To avoid incorrect associations between simultaneously occurring but causally independent events, candidate relationships are further filtered by considering temporal intervals, topology distances, anomaly compatibility, and log semantics. After event chain construction, root-cause nodes, propagation edges, key sensor segments, log contents, and operation records are converted into structured evidence packages and transmitted to the cloud-side reasoning module. The large language model is restricted to generating anomaly explanations, impact assessments, mitigation suggestions, and responsibility-oriented evidence descriptions based only on the provided evidence packages. Each generated statement is required to correspond to specific devices, timestamps, and evidence sources. When multiple evidence sources provide consistent support, comprehensive explanations can be generated; otherwise, when evidence is insufficient, reliability is low, or conflicts exist among different sources, an uncertainty rejection mechanism is activated to provide conservative judgments and indicate additional required evidence. Through this design, the three modules form a continuous processing pipeline from raw state representation, device-level anomaly identification, system-level root-cause analysis, to trustworthy explanation generation, thereby establishing traceable connections between low-level sensing results and high-level reasoning outputs.

#### 3.3.2. Trusted Multi-Sensor Temporal Encoding Module

The trusted multi-sensor temporal encoding module takes hardware operation state tensors within fixed temporal windows as inputs.

As shown in Figure 2, the input tensor is defined as $X\in R^{H_{0}\times W_{0}\times C_{0}}$, where $H_{0}$ denotes the number of sensor groups or device components, $W_{0}$ represents the temporal window length, and $C_{0}$ indicates the number of feature channels containing observations, missing indicators, temporal intervals, and statistical states. The network consists of $L_{c}$ trusted temporal convolution blocks, $L_{t}$ cross-channel Transformer layers, and two task-specific output branches. The input and output dimensions of the *l*-th convolution block are represented as $H_{l-1}\times W_{l-1}\times C_{l-1}$ and $H_{l}\times W_{l}\times C_{l}$, respectively. Since convolution operations are performed only along the temporal dimension, $H_{l}=H_{l-1}$ is generally maintained, while the temporal width is determined by the convolution kernel size $K_{l}$, stride $S_{l}$, dilation rate $D_{l}$, and padding size $P_{l}$:

(20) $$W_{l}=\left\lfloor \frac{W_{l-1}+2P_{l}-D_{l}\left(K_{l}-1\right)-1}{S_{l}}+1\right\rfloor .$$

To simultaneously capture short-term variations and long-term accumulated effects, each convolution block adopts dilated causal convolution, gated activation, and residual mapping. The intermediate representation of the *l*-th layer is formulated as:

(21) $$U_{l}=\tanh\left(C_{l}^{\left(f\right)}\left(Z_{l-1}\right)\right)⊙\sigma \left(C_{l}^{\left(g\right)}\left(Z_{l-1}\right)\right),$$

(22) $$Z_{l}=\mathrm{LN}\left(P_{l}Z_{l-1}+C_{l}^{\left(o\right)}\left(U_{l}\right)\right),$$

where $C_{l}^{\left(f\right)}$, $C_{l}^{\left(g\right)}$, and $C_{l}^{\left(o\right)}$ represent feature convolution, gated convolution, and output projection operations, respectively. $P_{l}$ is used for channel alignment when $C_{l-1}\neq C_{l}$. The dilation rate is gradually increased with network depth, allowing the receptive field of the *l*-th layer to be expressed as:

(23) $$R_{l}=1+\sum_{j=1}^{l}\left(K_{j}-1\right)\prod_{q=1}^{j}D_{q},$$

enabling deep features to cover long temporal dependencies without substantially increasing model parameters. This property is beneficial for identifying slowly accumulated temperature increases, continuous power elevation, and progressive network congestion patterns. The convolutional features are subsequently reshaped into a sequence with length $N=H_{L_{c}}W_{L_{c}}$ and projected into the embedding dimension $C_{e}$. The cross-channel Transformer consists of $L_{t}$ layers, where each layer contains *M* attention heads and the dimension of each head is $C_{e}/M$. The output of the *m*-th attention head is calculated as:

(24) $$A_{m}=\mathrm{Softmax}\left(\frac{Q_{m}K_{m}^{T}}{\sqrt{C_{e}/M}}+B_{\mathrm{topo}}+B_{\mathrm{time}}\right)V_{m},$$

where $B_{\mathrm{topo}}$ encodes device-level relationships among sensors, and $B_{\mathrm{time}}$ represents relative temporal distances. This design enables joint modeling of coupled variations among GPU temperature, power consumption, fan speed, and cabinet environmental conditions rather than independent analysis of individual signals. For a window of length *L*, the estimator calculates four normalized quality factors for channel *i*: completeness $c_{i}$, local stability $s_{i}$, freshness $d_{i}$, and cross-channel agreement $a_{i}$. They are implemented as

(25) $$\begin{array}{cccc} c_{i} & =\frac{1}{L}\sum_{t=1}^{L}m_{i}\left(t\right), & s_{i} & =\exp\;\left[-\frac{\mathrm{MAD}(\Delta x_{i})}{\kappa_{s}}\right],\\ d_{i} & =\exp\;\left[-\frac{1}{L\tau_{d}}\sum_{t=1}^{L}\delta_{i}\left(t\right)\right], & a_{i} & =\exp\;\left[-\frac{1}{|C_{i}|}\sum_{j\in C_{i}}\frac{∥{\tilde{x}}_{i}-{\tilde{x}}_{j}{∥}_{1}}{L\kappa_{a}}\right]. \end{array}$$

Here, $\mathrm{MAD}(\Delta x_{i})$ is the median absolute deviation of first differences, $C_{i}$ contains physically related channels, and $\kappa_{s}$, $\kappa_{a}$, and $\tau_{d}$ are computed from training-set robust scales. The vector $q_{i}={\left[c_{i},s_{i},d_{i},a_{i}\right]}^{T}$ is passed to a two-layer perceptron. A sigmoid bounds the reliability score in [0,1]:

(26) $$r_{i}=\sigma \left[w_{2}^{T}\phi \left(W_{1}q_{i}+b_{1}\right)+b_{2}\right],$$

The target $r_{i}^{*}$ is 1 for a valid clean channel and decreases with the recorded severity of missingness, noise, delay, drift, or freezing. For physics-consistent augmented windows, the corruption operator supplies this severity exactly; for observed acquisition faults, monitor status and the adjudicated acquisition-error label supply it. The reliability estimator is trained jointly with the anomaly task using

(27) $$L_{\mathrm{rel}}=-\frac{1}{N_{s}}\sum_{i=1}^{N_{s}}\left[r_{i}^{*}\log r_{i}+\left(1-r_{i}^{*}\right)\log\left(1-r_{i}\right)\right].$$

Consequently, $r_{i}$ represents the learned probability that channel *i* is usable within the current window rather than an unconstrained attention coefficient. The normalization below converts these probabilities into fusion weights.

(28) $$\alpha_{i}=\frac{r_{i}}{\sum_{j=1}^{H_{0}}r_{j}+\varepsilon },\;z=\sum_{i=1}^{H_{0}}\alpha_{i}h_{i},$$

where $h_{i}$ denotes the temporal representation of the *i*-th sensor and $\alpha_{i}$ represents the normalized reliability weight. The final state representation z is then delivered to the anomaly classification branch and temporal localization branch:

(29) $$\hat{y}=\mathrm{Softmax}\left(W_{a}z+b_{a}\right),\;\hat{\tau }=\sigma \left(W_{\tau }z+b_{\tau }\right).$$

The proposed reliability-weighted fusion mechanism provides an explicit robustness guarantee. Assume that the representation of the *i*-th sensor is disturbed by $\delta_{i}$, then the variation of the fused representation can be expressed as:

(30) $$\Delta z=\sum_{i=1}^{H_{0}}\alpha_{i}\delta_{i}.$$

According to the triangle inequality, it can be obtained that:

(31) $${∥\Delta z∥}_{2}\leq \sum_{i=1}^{H_{0}}\alpha_{i}{∥\delta_{i}∥}_{2}\leq \max_{i}{∥\delta_{i}∥}_{2}.$$

Furthermore, assuming that the corrupted sensor set is denoted as Ω, and the reliability estimation satisfies $\sum_{i\in \Omega }\alpha_{i}\leq \eta$, the representation perturbation is bounded by:

(32) $${∥\Delta z∥}_{2}\leq \eta \max_{i\in \Omega }{∥\delta_{i}∥}_{2}.$$

Therefore, when missing, drifting, or frozen sensor channels are assigned lower reliability weights, their influence on the overall representation can be constrained proportionally to η. Compared with fixed-weight fusion strategies, the proposed module is capable of distinguishing genuine device anomalies from sensor failures and maintaining stable anomaly recognition under noisy, asynchronous, and partially unavailable multi-source sensing conditions. Meanwhile, the extracted channel importance, anomaly probability, and temporal localization information can be directly transferred to the subsequent event chain construction module, providing interpretable low-level evidence for root-cause analysis and traceability.

#### 3.3.3. Cross-Device Anomaly Event Chain Construction Module

The cross-device module receives device-level anomaly events from the temporal encoder. It combines them with topology, log semantics, and task dependencies to localize system-level root causes.

As shown in Figure 3, the input event tensor is defined as $E_{0}\in R^{H_{0}\times W_{0}\times C_{0}}$, where $H_{0}$ represents the number of devices, $W_{0}$ denotes the number of event positions retained for each device within the analysis window, and $C_{0}$ represents feature channels including anomaly probabilities, occurrence timestamps, sensor reliability scores, state representations, and log embeddings. Valid events are expanded into *N* graph nodes and projected into initial node embeddings $H^{\left(0\right)}\in R^{N\times C_{g}}$ through linear transformation. The module consists of $L_{g}$ relational temporal graph encoder layers, a root-cause scoring branch, and a propagation relation prediction branch. Physical connections, network communications, service invocations, and task migrations among devices are represented as *R* types of relational adjacency tensors $A\in {\left\{0,1\right\}}^{R\times N\times N}$. In the *l*-th layer, neighborhood information under different relations is aggregated separately:

(33) $$m_{i}^{\left(l,r\right)}=\sum_{j\in N_{r}\left(i\right)}\gamma_{ij}^{\left(l,r\right)}W_{r}^{\left(l\right)}h_{j}^{\left(l\right)},$$

where $W_{r}^{\left(l\right)}\in R^{C_{l}\times C_{l+1}}$ represents the relation-specific feature transformation matrix, and $\gamma_{ij}^{\left(l,r\right)}$ is determined by temporal intervals, topology distances, anomaly compatibility, and log semantics:

(34) $$\gamma_{ij}^{\left(l,r\right)}=\frac{\exp\left(a_{r}^{(l)T}\psi \left[U_{r}^{\left(l\right)}h_{i}^{\left(l\right)}∥V_{r}^{\left(l\right)}h_{j}^{\left(l\right)}∥d_{ij}\right]\right)}{\sum_{k\in N_{r}\left(i\right)}\exp\left(a_{r}^{(l)T}\psi \left[U_{r}^{\left(l\right)}h_{i}^{\left(l\right)}∥V_{r}^{\left(l\right)}h_{k}^{\left(l\right)}∥d_{ik}\right]\right)}.$$

where $d_{ij}$ contains relative time information, device distance, invocation direction, and log matching degree. Different relational messages are further integrated through a gated mechanism to prevent incorrect associations between physically adjacent but functionally unrelated devices:

(35) $$g_{i}^{\left(l\right)}=\sigma \left(W_{g}^{\left(l\right)}\left[h_{i}^{\left(l\right)}∥\sum_{r=1}^{R}m_{i}^{\left(l,r\right)}\right]+b_{g}^{\left(l\right)}\right),$$

(36) $$h_{i}^{\left(l+1\right)}=g_{i}^{\left(l\right)}⊙\phi \left(\sum_{r=1}^{R}m_{i}^{\left(l,r\right)}\right)+\left(1-g_{i}^{\left(l\right)}\right)⊙S^{\left(l\right)}h_{i}^{\left(l\right)}.$$

After $L_{g}$ propagation layers, node representations are progressively transformed from $N\times C_{g}$ to $N\times C_{L_{g}}$. The root-cause branch estimates the probability of each node being the abnormal origin by jointly considering intrinsic abnormal intensity, outgoing propagation capability, and incoming dependency degree:

(37) $$p_{i}^{\mathrm{root}}=\frac{\exp\left(w_{r}^{T}h_{i}^{\left(L_{g}\right)}-\lambda_{\mathrm{in}}d_{i}^{\mathrm{in}}+\lambda_{\mathrm{out}}d_{i}^{\mathrm{out}}\right)}{\sum_{j=1}^{N}\exp\left(w_{r}^{T}h_{j}^{\left(L_{g}\right)}-\lambda_{\mathrm{in}}d_{j}^{\mathrm{in}}+\lambda_{\mathrm{out}}d_{j}^{\mathrm{out}}\right)}.$$

The propagation relation branch performs bilinear scoring between candidate node pairs and applies a temporal mask to prevent later-occurring events from pointing to earlier-occurring events:

(38) $$p_{ij}^{\mathrm{edge}}=\sigma \left(h_{i}^{(L_{g})T}W_{e}h_{j}^{\left(L_{g}\right)}+u_{e}^{T}d_{ij}\right)I\left(t_{i}<t_{j}\right).$$

The final anomaly event chain is obtained by jointly optimizing root-cause classification, relation prediction, and path consistency constraints:

(39) $$L_{\mathrm{chain}}=L_{\mathrm{root}}+\beta_{1}L_{\mathrm{edge}}+\beta_{2}L_{\mathrm{path}}+\beta_{3}L_{\mathrm{sparse}}.$$

The proposed graph construction mechanism satisfies node permutation equivariance. Let P denote an arbitrary device event permutation matrix. The reordered node features and adjacency relationships are represented as ${\tilde{H}}^{\left(0\right)}=PH^{\left(0\right)}$ and ${\tilde{A}}_{r}=PA_{r}P^{T}$, respectively. Since each graph layer performs shared parameter transformation and neighborhood aggregation, the following property can be obtained through mathematical induction:

(40) $${\tilde{H}}^{\left(l\right)}=PH^{\left(l\right)},\;l=1,\ldots ,L_{g}.$$

Therefore, changes in device indexing do not affect the structural inference of anomaly event chains. Furthermore, since all predicted propagation edges satisfy $t_{i}<t_{j}$, the existence of a directed cycle would require a strictly increasing temporal sequence that eventually returns to the initial event, resulting in the contradiction $t_{1}<t_{2}<\cdots <t_{1}$. Consequently, the generated event graph is guaranteed to be a directed acyclic graph. This module enables discrimination between root-cause devices and passively affected devices, while organizing distributed temperature, power, network, and log anomalies into traceable propagation chains. The generated structured evidence provides explicit device identities, temporal information, and relational dependencies for subsequent large language model reasoning.

#### 3.3.4. Evidence-Constrained LLM Reasoning Module

The evidence-constrained large language model reasoning module receives root-cause nodes, propagation edges, critical sensor segments, system logs, and operation records generated by the cross-device anomaly event chain construction module. These heterogeneous information sources are organized into a unified structured evidence tensor $S\in R^{H_{e}\times W_{e}\times C_{e}}$, where $H_{e}$ denotes the number of evidence entries, $W_{e}$ represents the maximum sequence width of each evidence item, and $C_{e}$ indicates feature channels including device identifiers, temporal information, anomaly categories, reliability scores, relationship types, and textual semantics. The module consists of $L_{e}$ evidence encoding layers, $L_{a}$ evidence interaction layers, $L_{d}$ language decoding layers, and an uncertainty estimation branch.

The practical information flow in Figure 4 has five steps. First, each retained sensor segment, event node, graph edge, log, alarm, and ticket is serialized with a source identifier, device identifier, time interval, content, and reliability field. Second, the encoder maps these records to ${\bar{e}}_{i}$. Third, the decoder state queries the records and multiplies semantic relevance by evidence reliability to obtain $g_{t,i}$. Fourth, the weighted context $c_{t}$ conditions the next-token logits, while $m_{t}$ blocks unsupported claim-bearing tokens. Fifth, sentence coverage, distribution entropy, and pairwise evidence conflict produce $u_{k}$; a sentence is emitted only when this rejection probability remains below the threshold.

As shown in Figure 4, structured fields and textual records are first projected into a shared representation space with dimension $C_{m}$ through field embedding and text embedding matrices. Evidence source embeddings, device position embeddings, and relative temporal embeddings are further incorporated to construct the initial evidence representation:

(41) $$E^{\left(0\right)}=SW_{p}+E_{\mathrm{src}}+E_{\mathrm{dev}}+E_{\mathrm{time}}.$$

The *l*-th evidence encoder layer adopts feed-forward transformation, residual connection, and normalization operations, transforming the input dimension from $H_{e}\times W_{e}\times C_{l}$ to $H_{e}\times W_{e}\times C_{l+1}$:

(42) $$E^{\left(l+1\right)}=\mathrm{Norm}\left(E^{\left(l\right)}+\phi \left(E^{\left(l\right)}W_{1}^{\left(l\right)}+b_{1}^{\left(l\right)}\right)W_{2}^{\left(l\right)}\right).$$

The reliability attached to evidence item *i* depends on its provenance. For a sensor segment it is the mean channel reliability; for a root node it is the product of graph root confidence and mean supporting-channel reliability; for a propagation edge it is the edge confidence times the geometric mean of its endpoint reliabilities; and for a log, alarm, or ticket it is the product of source-integrity, timestamp-match, and device-identifier-match scores:

(43) $$r_{i}^{\mathrm{evi}}=\left\{\begin{array}{cc} |I_{i}{|}^{-1}\sum_{j\in I_{i}}r_{j}, & i\;\mathrm{is}\;a\;\mathrm{sensor}\;\mathrm{segment},\\ p_{i}^{\mathrm{root}}{\bar{r}}_{i}, & i\;\mathrm{is}\;a\;\mathrm{root}\;\mathrm{node},\\ p_{ab}^{\mathrm{edge}}\sqrt{{\bar{r}}_{a}{\bar{r}}_{b}}, & i=(a,b)\;\mathrm{is}\;\mathrm{an}\;\mathrm{event}\;\mathrm{edge},\\ q_{i}^{\mathrm{src}}q_{i}^{\mathrm{time}}q_{i}^{\mathrm{dev}}, & i\;\mathrm{is}\;a\;\log,\;\mathrm{alarm},\;\mathrm{or}\;\mathrm{ticket}. \end{array}\right.$$

All factors lie in [0,1]. The time-match score is $q_{i}^{\mathrm{time}}=\exp(-|\Delta t_{i}\left|/\tau_{e}\right)$, and the source- and device-match scores are obtained from integrity checks and identifier resolution during preprocessing. Thus, the evidence reliability score has a traceable operational meaning. To select evidence at decoding position *t*, let $q_{t}$ denote the language state and ${\bar{e}}_{i}$ the representation of evidence item *i*. The gate is

(44) $$g_{t,i}=\sigma \left(q_{t}^{T}W_{g}{\bar{e}}_{i}+b_{g}\right)r_{i}^{\mathrm{evi}},$$

so an item contributes only when it is both semantically relevant and operationally reliable. The evidence-aware context is

(45) $$c_{t}=\frac{\sum_{i=1}^{H_{e}}g_{t,i}{\bar{e}}_{i}}{\sum_{i=1}^{H_{e}}g_{t,i}+\epsilon }.$$

The language decoder jointly considers the current language state, evidence context, and generated tokens to predict the next token distribution:

(46) $$p\left(y_{t}|y_{<t},S\right)=\mathrm{Softmax}\left(W_{o}\left[q_{t}∥c_{t}\right]+b_{o}+m_{t}\right),$$

The mask applies to claim-bearing tokens rather than grammatical function words. Let V(S) contain device identifiers, timestamps, values, anomaly types, relation predicates, and actions supported by the evidence package; $V_{\mathrm{func}}$ contains grammatical tokens; and $V_{\mathrm{unc}}$ contains approved uncertainty phrases. The implementation is

(47) $$m_{t,v}=\left\{\begin{array}{cc} 0, & v\in V\left(S\right)\cup V_{\mathrm{func}}\cup V_{\mathrm{unc}},\\ -\infty , & v\;\mathrm{is}\;\mathrm{an}\;\mathrm{unsupported}\;\mathrm{entity},\;\mathrm{value},\;\mathrm{or}\;\mathrm{causal}\;\mathrm{predicate}. \end{array}\right.$$

This construction preserves fluent connecting text but prevents an unseen device, time, measurement, or deterministic causal relation from entering the output. For anomaly causes, impact analysis, mitigation recommendations, and responsibility-oriented evidence descriptions, the model further estimates sentence-level evidence coverage:

(48) $$s_{k}=\frac{1}{|Y_{k}|}\sum_{t\in Y_{k}}\max_{i}g_{t,i},$$

Here, $Y_{k}$ is the token set of sentence *k*. Pairwise conflict is predicted between evidence items that refer to the same device and overlapping time interval. With ${\hat{c}}_{ij}\in \left[0,1\right]$ denoting contradiction probability, conflict degree is the reliability-weighted mean

(49) $$\chi_{k}=\frac{\sum_{i<j}r_{i}^{\mathrm{evi}}r_{j}^{\mathrm{evi}}{\hat{c}}_{ij}}{\sum_{i<j}r_{i}^{\mathrm{evi}}r_{j}^{\mathrm{evi}}+\epsilon }.$$

When coverage is insufficient or reliable sources conflict, the uncertainty branch estimates the rejection probability

(50) $$u_{k}=\sigma \left(w_{u}^{T}\left[1-s_{k},\mathrm{Ent}\left(p_{k}\right),\chi_{k}\right]+b_{u}\right),$$

where $\mathrm{Ent}(p_{k})$ is generation entropy. The rejection threshold is $\tau_{u}=0.60$, selected on the validation set to maximize correct-rejection F1 and then held fixed for testing. When $u_{k}>\tau_{u}$, the decoder replaces deterministic attribution with an insufficient- or conflicting-evidence statement and lists the missing record types.

The loss terms are implemented as follows. $L_{\mathrm{gen}}$ is next-token negative log-likelihood. If $a_{t}^{*}$ is the annotated supporting evidence item for target token $y_{t}^{*}$, $L_{\mathrm{align}}$ is the negative log normalized gate assigned to that item. With pairwise contradiction label $c_{ij}^{*}$ and sentence rejection label $z_{k}^{*}$, the conflict and uncertainty losses are binary cross-entropy and Brier calibration loss, respectively:

(51) $$L_{\mathrm{gen}}=-\frac{1}{T}\sum_{t}\log p\left(y_{t}^{*}|y_{<t}^{*},S\right),\;L_{\mathrm{align}}=-\frac{1}{T}\sum_{t}\log\frac{g_{t,a_{t}^{*}}}{\sum_{i}g_{t,i}+\epsilon },$$

(52) $$L_{\mathrm{conflict}}=-\frac{1}{\left|P\right|}\sum_{(i,j)\in P}\left[c_{ij}^{*}\log{\hat{c}}_{ij}+\left(1-c_{ij}^{*}\right)\log\left(1-{\hat{c}}_{ij}\right)\right],\;L_{\mathrm{uncertainty}}=\frac{1}{K}\sum_{k=1}^{K}{\left(u_{k}-z_{k}^{*}\right)}^{2}.$$

The overall reasoning objective is

(53) $$L_{\mathrm{reason}}=L_{\mathrm{gen}}+\lambda_{1}L_{\mathrm{align}}+\lambda_{2}L_{\mathrm{conflict}}+\lambda_{3}L_{\mathrm{uncertainty}}.$$

The weights are $\lambda_{1}=0.5$, $\lambda_{2}=0.2$, and $\lambda_{3}=0.2$. They are selected on the validation set and remain fixed in every test and ablation run. The evidence-constrained generation mechanism provides an explicit theoretical guarantee for suppressing unsupported statements. Let $V_{u}$ denote the set of all tokens without evidence support. For any token $v\in V_{u}$, the evidence mask satisfies $m_{t,v}\to -\infty$, and therefore:

(54) $$\lim_{m_{t,v}\to -\infty }p\left(y_{t}=v|y_{<t},S\right)=0.$$

Therefore, unsupported tokens cannot enter the final generated sequence under the evidence constraint. Furthermore, if the minimum evidence coverage of a generated sentence is no lower than η, the proportion of generation content without evidence grounding can be bounded as:

(55) $$\frac{1}{|Y_{k}|}\sum_{t\in Y_{k}}\left(1-\max_{i}g_{t,i}\right)\leq 1-\eta .$$

This property indicates that the uncertainty of generated explanations decreases as evidence coverage increases. The proposed module constrains large language models within structured evidence boundaries while preserving their ability to summarize complex events and generate operational recommendations. Compared with directly providing raw logs or sensor sequences to general-purpose large language models, the proposed evidence-constrained design effectively reduces unsupported reasoning and excessive deterministic attribution. Meanwhile, the generated anomaly causes, propagation impacts, and responsibility-oriented auxiliary descriptions remain traceable to specific devices, timestamps, and evidence sources, enabling trustworthy reasoning for complex hardware security scenarios.

## 4. Results and Discussion

### 4.1. Experimental Configuration

#### 4.1.1. Hardware and Software Platform

All experiments were conducted on a server platform equipped with an Intel Xeon Gold 6338 processor, 256 GB of DDR4 memory, and an NVIDIA RTX 4090 GPU with 24 GB of video memory. The data acquisition environment consisted of servers, switches, edge computing nodes, and terminal devices. NVIDIA Jetson AGX Orin platforms with 64 GB of shared memory were deployed as edge nodes to support sensor data access, real-time preprocessing, and edge-side anomaly detection. The experimental environment was also connected to monitoring devices for temperature, power consumption, current, voltage, fan speed, cabinet temperature and humidity, and network operating states. Data transmission among devices was performed through Gigabit Ethernet. To ensure the stability and fairness of the experimental results, all models were trained and evaluated under identical computing platforms and resource constraints. Training time, inference latency, GPU memory consumption, and energy consumption were recorded separately for each model. Ubuntu 22.04 LTS was adopted as the operating system, and all models were implemented using Python 3.10 and PyTorch 2.3. GPU acceleration was supported by CUDA 12.1 and cuDNN 8.9. NumPy, Pandas, SciPy, and Scikit-learn were mainly used for data preprocessing and statistical analysis, while tsfresh and PyWavelets were employed for time-series processing. Device topology modeling and anomaly event graph construction were implemented using NetworkX and PyTorch Geometric. The large language model reasoning module was developed based on Transformers and LangChain. TensorBoard was used to record training losses and evaluation metrics, while Optuna was employed for partial hyperparameter optimization. Experimental reproducibility was improved by fixing software dependency versions, random seeds, and runtime environment configurations.

The dataset was chronologically divided into training, validation, and test sets at ratios of 70%, 15%, and 15%, respectively, thereby preventing data leakage caused by adjacent temporal segments appearing simultaneously in the training and test sets. The training set was used for model parameter learning, the validation set was used for hyperparameter selection and early-stopping decisions, and the test set was used exclusively for final performance evaluation. In cross-device experiments, data were partitioned at the device level to ensure that data from test devices were not included during model training. Five-fold cross-validation was additionally conducted on the training data. Specifically, the training set was divided into five mutually exclusive subsets, of which four were used for training and the remaining subset was used for validation in each fold. The mean results and corresponding 95% confidence intervals over the five folds were ultimately reported. All deep learning models were trained using the AdamW optimizer. The initial learning rate was set to $1\times 10^{-4}$, the weight decay coefficient was set to $1\times 10^{-5}$, the batch size was set to 64, and the maximum number of training epochs was set to 100. Training was terminated early when the validation loss failed to decrease for 10 consecutive epochs, and the model parameters associated with the best validation performance were retained. A cosine annealing schedule was adopted for learning-rate adjustment, the gradient clipping threshold was set to 1.0, and the dropout rate was set to 0.2. The input window length was set to 128 sampling points, the sliding stride was set to 32, and the hidden feature dimension was set to 128. The LSTM baseline contained two recurrent layers, whereas the TCN baseline consisted of four dilated convolutional blocks with a kernel size of three. Both the Transformer and Cross-modal Transformer were configured with four encoder layers and eight attention heads, and the feed-forward network dimension was set to 512. For PatchTST, the patch length and stride were set to 16 and eight, respectively. The graph neural network employed a three-layer message-passing architecture. For Random Forest, the number of decision trees was set to 300, and the maximum number of features was determined using the square-root strategy. For XGBoost, the number of trees was set to 500, the maximum tree depth was set to six, the learning rate was set to 0.05, and the subsampling ratio was set to 0.8. A fixed random seed of 42 was used in all experiments, and each experimental configuration was independently repeated five times to reduce the influence of random initialization.

#### 4.1.2. Baseline Models and Evaluation Metrics

Random Forest [34], XGBoost [35], LSTM [36], TCN [37], Transformer [38], PatchTST [39], Early Fusion [40], Cross-modal Transformer [41], and Graph Neural Network [42] were selected as baseline methods. Random Forest performs anomaly identification by constructing multiple decision trees and aggregating their predictions, providing strong adaptability to nonlinear features and noisy data. XGBoost progressively optimizes weak learners through a gradient boosting mechanism and offers high classification accuracy together with interpretable feature importance. LSTM employs gated recurrent structures to model long-term temporal dependencies in sensor data and alleviates the vanishing-gradient problem commonly encountered in conventional recurrent neural networks. TCN extracts both local and long-range temporal patterns through dilated causal convolutions, enabling parallel computation and efficient training. Transformer learns dependencies across time points and sensor channels through self-attention and provides strong global dependency modeling capability. PatchTST divides time-series data into local patches and encodes them independently, reducing the effective sequence length while preserving local variation patterns. Early fusion directly concatenates heterogeneous sensor features at the input stage and is characterized by a simple architecture and relatively low computational cost. The cross-modal transformer employs cross-modal attention to facilitate interactions among multi-source sensor measurements, logs, and device-state information, thereby capturing complementary relationships across modalities. The graph neural network represents devices and their interconnections as a graph structure, allowing system topology to be exploited for cross-device anomaly propagation analysis.

Accuracy, Precision, Recall, F1-score, and AUC were adopted to evaluate the classification performance of the hardware anomaly detection models. The evaluation metrics were calculated as follows:

(56) $$\mathrm{Accuracy}=\frac{TP+TN}{TP+TN+FP+FN},$$

(57) $$\mathrm{Precision}=\frac{TP}{TP+FP},$$

(58) $$\mathrm{Recall}=\frac{TP}{TP+FN},$$

(59) $$F1=\frac{2\times \mathrm{Precision}\times \mathrm{Recall}}{\mathrm{Precision}+\mathrm{Recall}},$$

(60) $$\mathrm{TPR}=\frac{TP}{TP+FN},\;\mathrm{FPR}=\frac{FP}{FP+TN},$$

(61) $$\mathrm{AUC}=\int_{0}^{1}\mathrm{TPR}\left(\mathrm{FPR}\right)\;d\mathrm{FPR}.$$

Here, TP denotes the number of anomalous samples correctly identified as anomalous, TN denotes the number of normal samples correctly identified as normal, FP denotes the number of normal samples incorrectly classified as anomalous, and FN denotes the number of anomalous samples incorrectly classified as normal. TPR and FPR denote the true-positive rate and false-positive rate, respectively.

### 4.2. Hardware Anomaly Detection Experiment

This experiment was designed to evaluate the ability of different models to identify hardware anomalies in cloud–edge–end environments and to compare static machine learning, temporal modeling, multi-source fusion, and topology-aware modeling methods in terms of classification accuracy, false-positive control, anomaly recall, and overall discriminative capability.

As shown in Table 3 and Figure 5, random forest achieved an Accuracy of 84.26%, an F1-score of 83.36%, and an AUC of 88.63%, indicating that fundamental anomaly patterns could be recognized, although complex temporal variations were not adequately characterized. The Accuracy obtained by XGBoost increased to 86.17%, while its F1-score reached 85.27%, demonstrating that nonlinear feature interactions were more effectively captured through gradient boosting. An Accuracy of 87.84% and an AUC of 91.76% were obtained by LSTM, confirming the importance of long-term temporal dependencies for hardware-state recognition. TCN achieved an Accuracy of 89.43% and an F1-score of 88.76%, thereby outperforming LSTM. The performance was further improved by Transformer, which achieved an Accuracy of 90.21% and an AUC of 93.91%. Through local patch-based temporal modeling, PatchTST increased the Accuracy and AUC to 91.37% and 94.82%, respectively. Early Fusion achieved an Accuracy of 89.76%. Although this result was superior to those obtained by several single-stream temporal models, it remained below that of PatchTST, suggesting that direct feature concatenation was insufficient to fully exploit complementary information across modalities. Cross-modal Transformer achieved an Accuracy of 92.48% and an AUC of 95.63%, indicating that cross-modal interactions enabled effective integration of sensing measurements, logs, and device-state information. Graph Neural Network was identified as the strongest baseline, with an Accuracy of 93.26%, an F1-score of 92.69%, and an AUC of 96.21%. The best performance across all metrics was achieved by the proposed method, with Accuracy, Precision, Recall, F1-score, and AUC values of 95.38%, 95.06%, 94.71%, 94.88%, and 97.41%, respectively. The narrowest confidence intervals were also obtained, indicating greater performance stability across independent runs. From a modeling perspective, Random Forest and XGBoost perform classification through piecewise decision boundaries in the feature space. Although nonlinear relationships can be represented, explicit constraints on sampling order and anomaly evolution are absent, making slowly developing faults, such as gradual temperature increases, power drift, and latency accumulation, difficult to identify. Historical information is recursively propagated through gated states in LSTM; however, serial computation and state compression may attenuate fine-grained variations over long temporal ranges. Dilated causal convolutions are employed by TCN to expand the receptive field, allowing local abrupt changes and long-term trends to be extracted in parallel, which accounts for its superiority over LSTM. The transformer directly estimates associations among arbitrary time points and sensor channels through attention weights, thereby reducing information attenuation over long dependency paths. PatchTST further partitions a sequence into local patches, allowing adjacent observations to form more stable local representations while reducing interference from redundant time points during attention computation. Early fusion performs only input-level feature concatenation, without explicitly accounting for differences in scale, reliability, or semantic meaning among heterogeneous data sources. Conditional dependencies among sensing measurements, logs, and device states can be learned by a cross-modal transformer through cross-modal attention, resulting in stronger performance. A graph neural network propagates node representations through adjacency relationships and is therefore well suited to identifying cross-device anomaly diffusion, although its effectiveness remains dependent on the quality of fixed topology and node features. In the proposed method, trustworthy temporal sensing encoding, dynamic reliability weighting, and cross-device event-chain modeling are jointly incorporated. Consequently, the influence of low-quality channels is suppressed, while temporal dependence, device topology, and anomaly propagation direction are represented within a unified framework. False positives are therefore reduced while anomaly recall is improved, leading to higher and more stable overall performance.

### 4.3. Root-Cause Localization and Anomaly Propagation Analysis Experiment

This experiment was designed to evaluate the joint capability of different models to identify the root causes of complex hardware anomalies, rank candidate root causes, recognize cross-device event relationships, and localize anomaly onset times. It was thereby determined whether anomaly detection could be extended toward an interpretable analysis of how an anomaly originated and propagated.

The purpose of Figure 6 is not to compare a percentage or MRR numerically with an error measured in seconds. Root-cause accuracy, Top-3 accuracy, MRR, and event-relation F1 quantify which device and relation are identified, whereas temporal localization error quantifies when the onset is identified. They are displayed together because they are complementary outputs of the same event-chain task, but each panel has its own vertical axis and optimization direction: higher is better for the first four panels and lower is better for the final panel.

As shown in Table 4 and Figure 6, random forest achieved a root-cause localization accuracy of 68.42%, an event relation F1-score of 65.83%, and a temporal localization error of 6.84 s, indicating that dynamic propagation processes could not be effectively reconstructed from static feature partitions. The root-cause localization accuracy obtained by XGBoost increased to 71.65%, while its MRR reached 0.778; however, cross-device dependencies remained insufficiently represented. LSTM achieved a root-cause localization accuracy of 74.38% and a temporal localization error of 5.48 s, demonstrating that historical state information contributed to root-cause identification. TCN further improved the localization accuracy to 77.91% and the event relation F1-score to 75.83%, outperforming LSTM. Root-cause localization accuracies of 79.46% and 81.73% were achieved by Transformer and PatchTST, respectively, while the temporal localization error of PatchTST was reduced to 3.91 s. Early fusion achieved an accuracy of 78.84%, which remained below that of PatchTST, indicating that simple concatenation was insufficient for extracting complex relational information. Cross-modal transformer increased the accuracy to 85.37%. Graph neural network further achieved a root-cause localization accuracy of 88.62%, an event relation F1-score of 87.46%, and a temporal localization error of 2.47 s. The proposed method achieved a root-cause localization accuracy of 91.84%, a Top-3 accuracy of 97.26%, an MRR of 0.944, an event relation F1-score of 90.73%, and a temporal localization error of only 1.82 s. The narrowest confidence intervals were also obtained, demonstrating more stable root-cause ranking and anomaly propagation analysis. From a modeling perspective, root causes are inferred by Random Forest and XGBoost through piecewise decision rules in the feature space. Although nonlinear variable interactions can be captured, temporal order and device-structure constraints are absent, making accompanying anomalies likely to be misidentified as root causes. In LSTM, historical information is compressed into recurrent states, allowing continuous changes before and after an anomaly to be represented; nevertheless, early signals in long propagation chains may gradually decay through repeated state updates. The temporal receptive field is expanded by TCN through dilated causal convolutions, while the forward direction of anomaly evolution is preserved, thereby yielding lower temporal localization errors than LSTM. Long-range relationships between events are directly established by Transformer through attention. However, highly correlated events may still be incorrectly interpreted as propagation relationships when topology constraints are unavailable. Local patch encoding in PatchTST reduces interference from short-term noise and facilitates recognition of localized variations during the initial stage of an anomaly. Early fusion only merges multi-source features at the input level and cannot dynamically distinguish the relative importance of heterogeneous evidence. Conditional cross-modal interactions are enabled by cross-modal transformer, substantially improving candidate root-cause ranking. Node information is propagated through the adjacency matrix in the graph neural network, making device dependencies suitable for graph-based modeling; however, a fixed graph structure cannot fully represent propagation directions that evolve over time. In the proposed method, trustworthy temporal representations, relational graph propagation, chronological constraints, and root-cause ranking are jointly modeled. Candidate root causes are therefore constrained simultaneously by event time, device connectivity, propagation direction, and evidence reliability. Spurious associations caused by shared workloads or synchronized fluctuations are consequently reduced, resulting in higher localization accuracy and lower temporal error.

### 4.4. Ablation Study

This ablation study was designed to quantify the individual contributions of the core components to hardware anomaly detection, root-cause localization, event relation recognition, and trustworthy explanation generation, while also evaluating the collaborative effects among different modules.

As shown in Table 5 and Figure 7, after sensor reliability estimation was removed, the anomaly detection F1-score decreased to 91.72%, while root-cause localization accuracy decreased to 87.36%, indicating that low-quality sensing channels interfered with system-state assessment. Removal of temporal synchronization and the missing-value mask further reduced the F1-score to 90.84% and increased the unsupported statement rate to 9.12%, demonstrating that temporal misalignment and confusion between imputed and genuine observations disrupted multi-source correspondence. When device topology modeling was removed, anomaly detection performance remained relatively high at 93.86%, whereas root-cause localization accuracy and event relation F1-score decreased substantially to 84.59% and 82.74%, respectively. This finding indicates that topology information contributed primarily to root cause and propagation analysis rather than basic anomaly classification. Removal of the anomaly event chain module resulted in the lowest event relation F1-score of 80.83%, while the evidence support rate decreased to 85.36%. Performance degradation across all tasks was observed after cloud–edge dynamic collaboration was removed, demonstrating that hierarchical computation contributed to the balance between real-time perception and complex reasoning. When the evidence constraint of the large language model was removed, only minor changes were observed in anomaly detection performance; however, the evidence support rate decreased sharply to 78.42%, and the unsupported statement rate increased to 18.63%. Removal of logs and operation records resulted in marked degradation in both root-cause localization and explanation quality. When the uncertainty rejection mechanism was removed, the unsupported statement rate increased to 11.92%. Removal of robust data augmentation produced a moderate but consistent decline across the evaluated metrics. The full model achieved an anomaly detection F1-score of 94.88%, a root-cause localization accuracy of 91.84%, an event relation F1-score of 90.73%, and an evidence support rate of 94.68%, while reducing the unsupported statement rate to 4.26%. The RAG-only baseline receives the same retrieved evidence package as plain prompt context but does not use reliability gates, the claim-bearing vocabulary mask, or uncertainty rejection. Relative to this standard retrieval-augmented decoder, the complete method increases evidence support from 78.42% to 94.68% (+16.26 percentage points) and reduces unsupported statements from 18.63% to 4.26% (−14.37 percentage points). Removing only uncertainty rejection raises unsupported statements from 4.26% to 11.92%. These controlled comparisons isolate the benefit of evidence control from the benefit of retrieval itself. From a modeling perspective, sensor reliability estimation suppresses missing, drifting, and noise-contaminated channels through dynamic weighting, thereby reducing the contribution of unreliable observations during feature aggregation. Temporal synchronization and missing-value masks establish a unified temporal coordinate system across devices and explicitly distinguish genuine observations from estimated values. Their removal therefore increases feature variance and temporal mismatch. Device topology modeling constrains information propagation through adjacency relationships, allowing root-cause candidates to be restricted by physical connections and service dependencies. Accordingly, its largest contribution was observed in root-cause localization and event relation recognition. The anomaly event chain module jointly represents event chronology, propagation direction, and node relationships, thereby reducing the likelihood that statistical correlation is incorrectly interpreted as a causal relationship. Through cloud–edge dynamic collaboration, computational tasks are allocated between local edge encoding and global cloud reasoning, allowing communication redundancy to be reduced while cross-device context is retained. The evidence constraint mechanism restricts the generation distribution according to retrieved evidence, making the large language model more likely to produce statements supported by sensing measurements, logs, and legal documents. The uncertainty rejection mechanism reduces generation confidence when evidence is insufficient, thereby suppressing unsupported conclusions. Logs and operation records provide operational semantics and event context, while robust augmentation broadens the training distribution to cover missingness, drift, and compound faults. In the full model, reliability, time, topology, event chains, and evidence constraints are jointly represented, resulting in the most stable overall performance.

### 4.5. Discussion

The results show that hardware anomaly detection in banking data-center IT operations can extend to root-cause localization, propagation analysis, and evidence-grounded interpretation. During month-end settlement or overnight batch processing, computational load, power consumption, and cabinet temperature often rise together. A threshold rule can mistake this normal peak for a failure. The proposed model instead combines processor load, fan speed, power state, environmental measurements, and task-queue change. It can therefore distinguish workload growth from a thermal-management fault. For example, a persistent fall in fan speed followed by local heating, processor throttling, and task delay supports the fan as the root cause rather than treating temperature as an isolated alarm. More direct practical value is provided in cross-device fault scenarios. For example, congestion at a core switch port may first be reflected by increases in packet-loss rate and queue length, followed by response delays at edge gateways, database connection timeouts, and transaction failures at business terminals. Through joint analysis of device topology, service invocation relationships, and event chronology, the anomaly propagation path can be traced from the switch port to the affected business nodes, thereby avoiding unnecessary inspection of database servers and terminal devices. When an anomaly coincides with a configuration change or manual operation, maintenance tickets, bastion-host records, and system logs can be incorporated to determine whether the incident is associated with misconfiguration, equipment maintenance, or access operations. Explanations generated by the cloud-side large language model are constrained by sensing measurements, logs, maintenance tickets, and regulatory materials. Consequently, incident investigation summaries containing the root-cause device, impact scope, operational basis, and responsibility-related evidence can be generated to support fault recovery, audit review, security operations, and business continuity management in banking data centers.

### 4.6. Limitations and Future Work

Several limitations remain. First, the data were primarily collected from the headquarters data center and same-city disaster recovery center of a single commercial bank. The equipment models, business workloads, and operation and maintenance procedures were therefore relatively homogeneous, and the generalization capability of the model across other financial institutions, heterogeneous cloud platforms, and different network architectures requires further validation. Second, only a limited number of complex compound faults and extreme security incidents were available. Anomaly labels were determined from alarms, fault tickets, and manual review, potentially introducing ambiguous boundaries and annotation bias. Device topology and service dependencies may also vary dynamically with configuration changes, while static relational information may not fully represent temporary task migration and service rerouting. Although legal documents can support responsibility-oriented interpretation, model-generated conclusions cannot replace professional legal judgment. Future research will be extended to multi-institutional, multi-data-center, and cross-regional disaster recovery environments. A larger dataset covering additional equipment types, business stages, and compound fault conditions will be constructed, and model stability will be evaluated through cross-device, cross-temporal, and cross-institutional validation. Dynamic topology learning, online incremental updating, and adaptive edge-model mechanisms will also be incorporated to improve responsiveness to configuration changes and previously unseen faults. Evidence retrieval, source verification, and uncertainty assessment mechanisms will be further strengthened so that generated statements can be explicitly aligned with sensing records, operation and maintenance logs, and regulatory provisions. Practical applicability will additionally be evaluated through joint assessment by operation and maintenance personnel, cybersecurity experts, and legal professionals.

## 5. Conclusions

This study presents a cloud–edge–end framework for trustworthy analysis of banking data-center IT operations. The data are hardware, network, system, environmental, alarm, maintenance, and topology records from a financial institution. The framework makes sensor quality an explicit variable and propagates it from temporal encoding to event-graph inference and evidence-constrained decoding. This coupling is the central technical contribution. It allows the model to suppress unreliable channels, distinguish root causes from downstream symptoms, and prevent unsupported device, time, and causal claims. The method achieves 95.38% Accuracy, a 94.88% F1-score, and a 97.41% AUC for anomaly detection. It also achieves 91.84% root-cause localization accuracy, 97.26% Top-3 Accuracy, a 90.73% event-relation F1-score, and a 1.82 s temporal localization error. Evidence support reaches 94.68%, while unsupported statements fall to 4.26%. The results support a traceable workflow from sensor observation to operational diagnosis, subject to the stated single-institution and data-availability limitations.

## Figures and Tables

**Figure 1:**
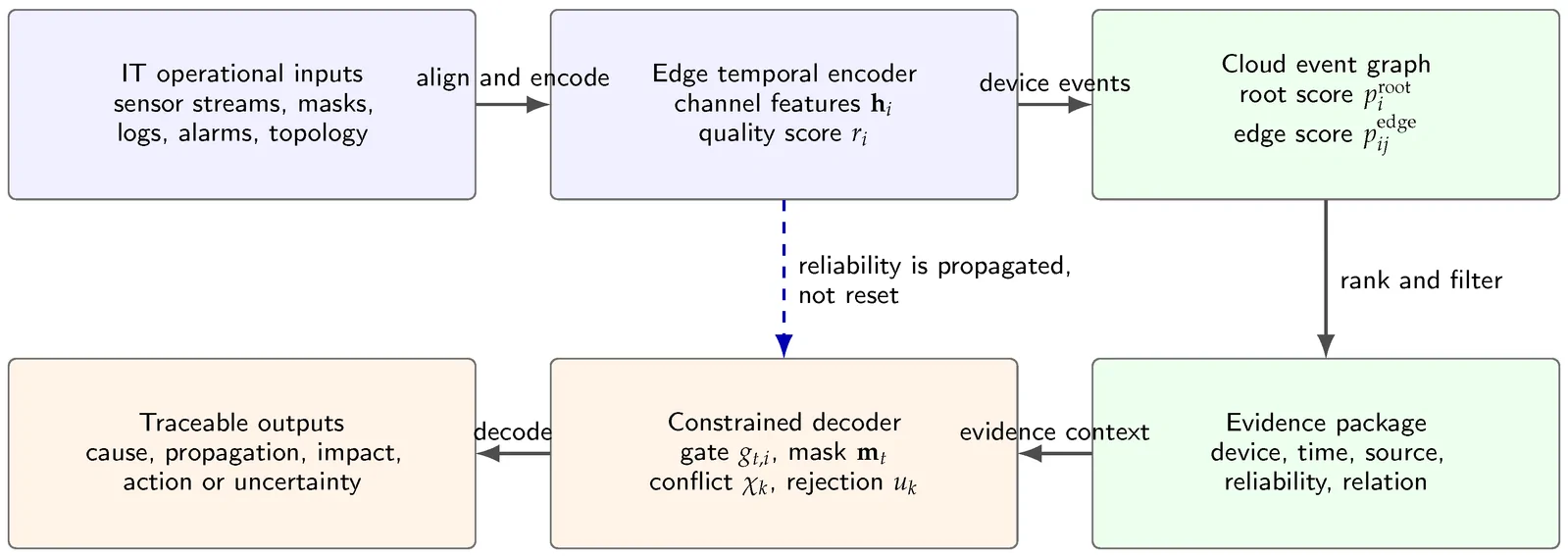
Overall prediction and evidence flow of the proposed reliability-propagation framework.

**Figure 2:**
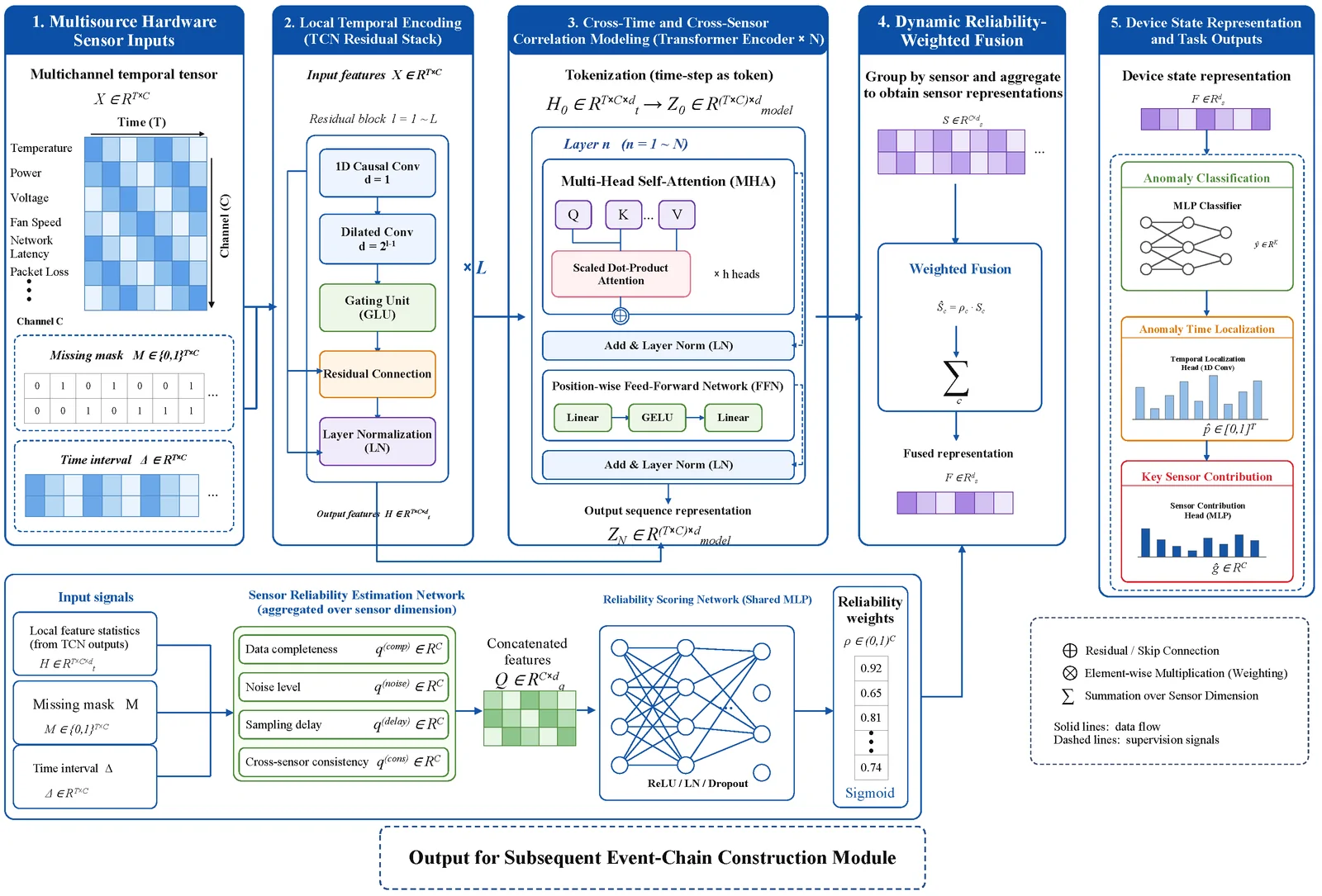
Dynamic reliability-weighted multi-sensor temporal encoder.

**Figure 3:**
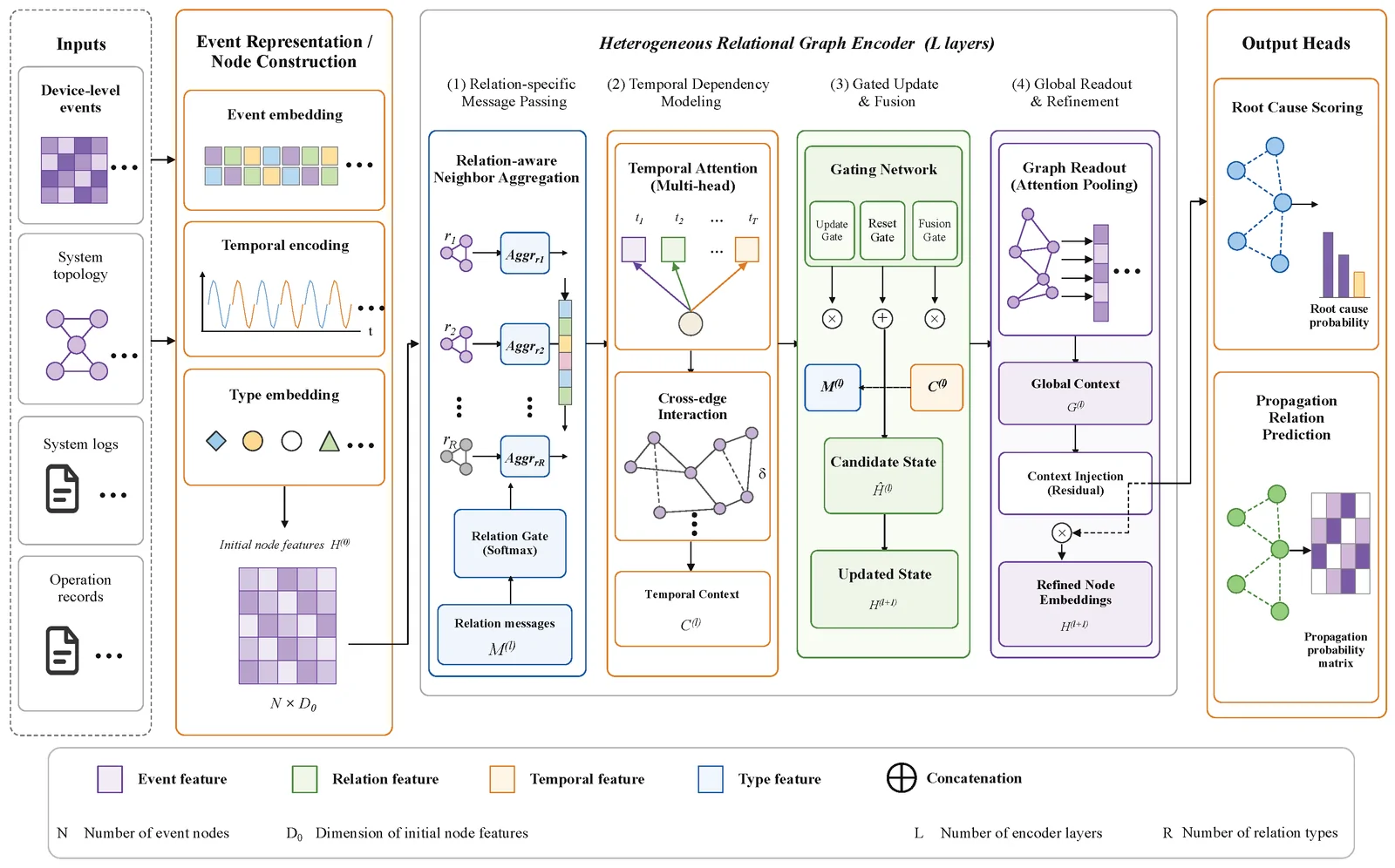
Topology- and chronology-constrained cross-device anomaly event graph.

**Figure 4:**
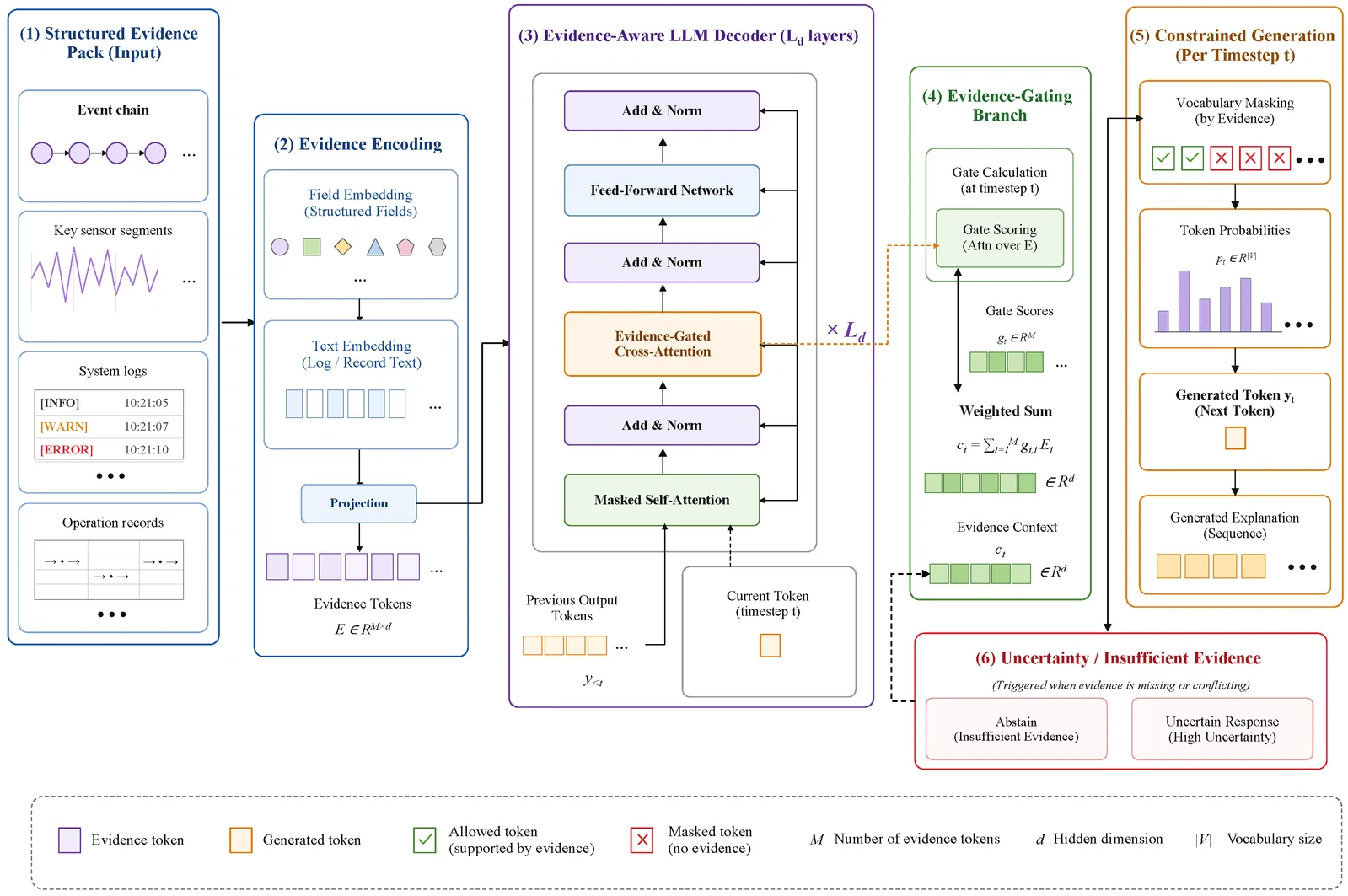
Evidence-gated decoding, vocabulary constraint, conflict estimation, and uncertainty rejection.

**Figure 5:**
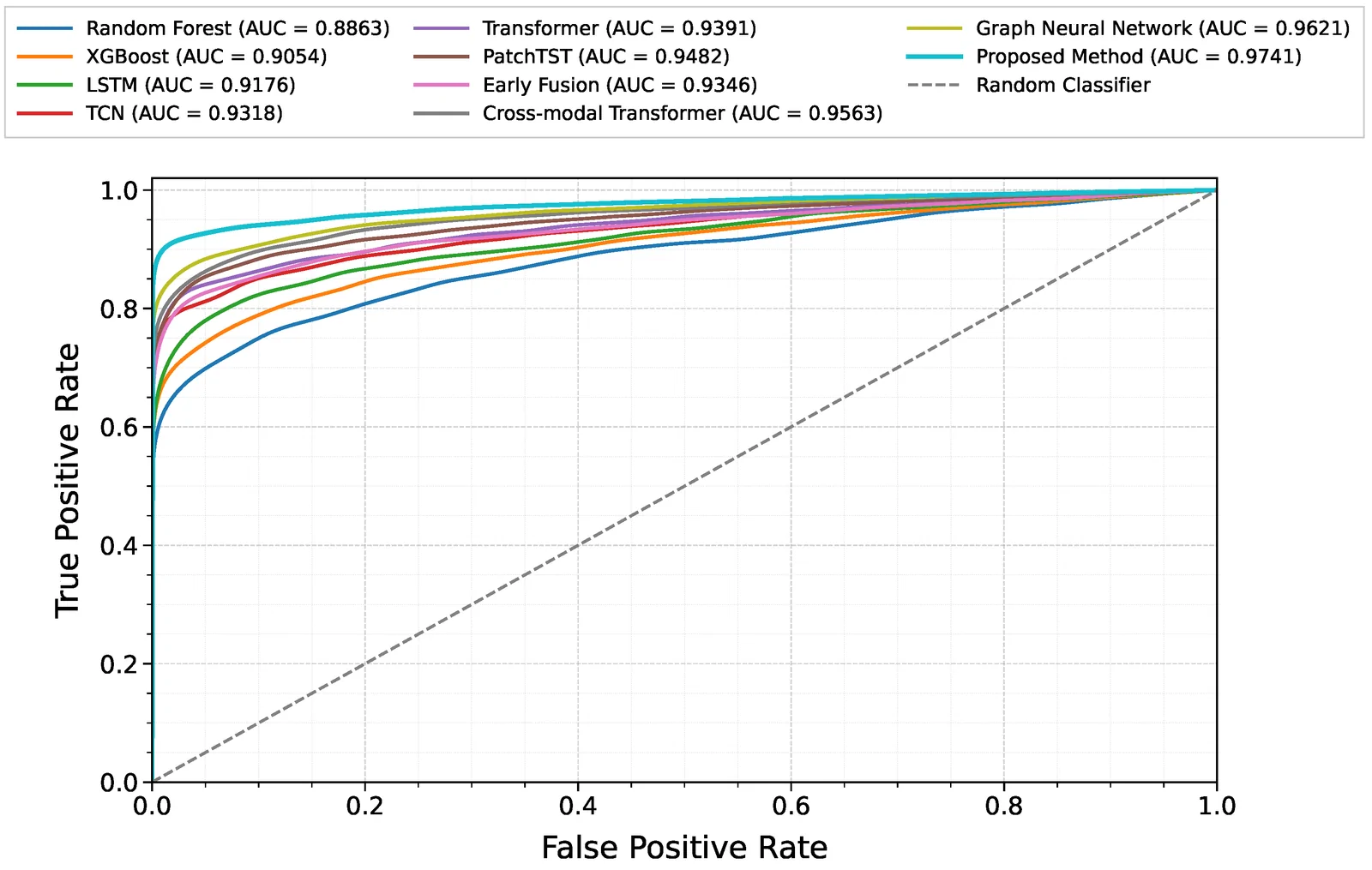
ROC curves of different methods for hardware anomaly detection, where the proposed method achieves the highest AUC of 0.9741 and demonstrates the strongest discrimination between anomalous and normal states.

**Figure 6:**
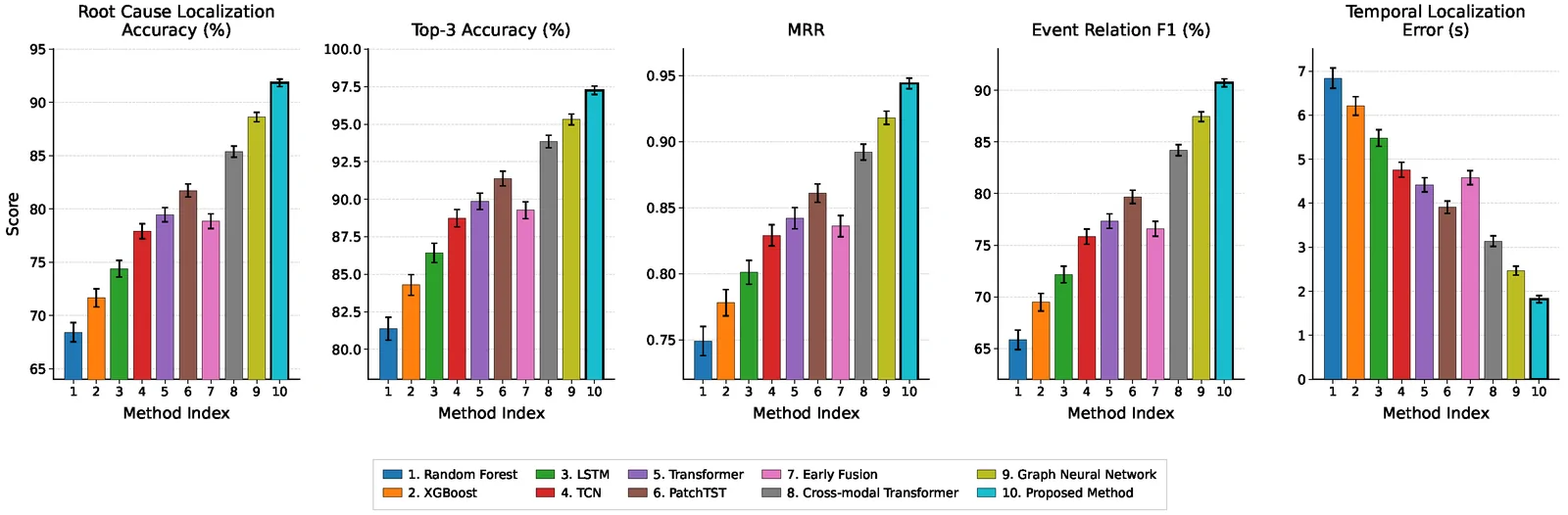
Root-cause, ranking, relation, and temporal-localization results shown in separate panels with metric-specific vertical axes.

**Figure 7:**
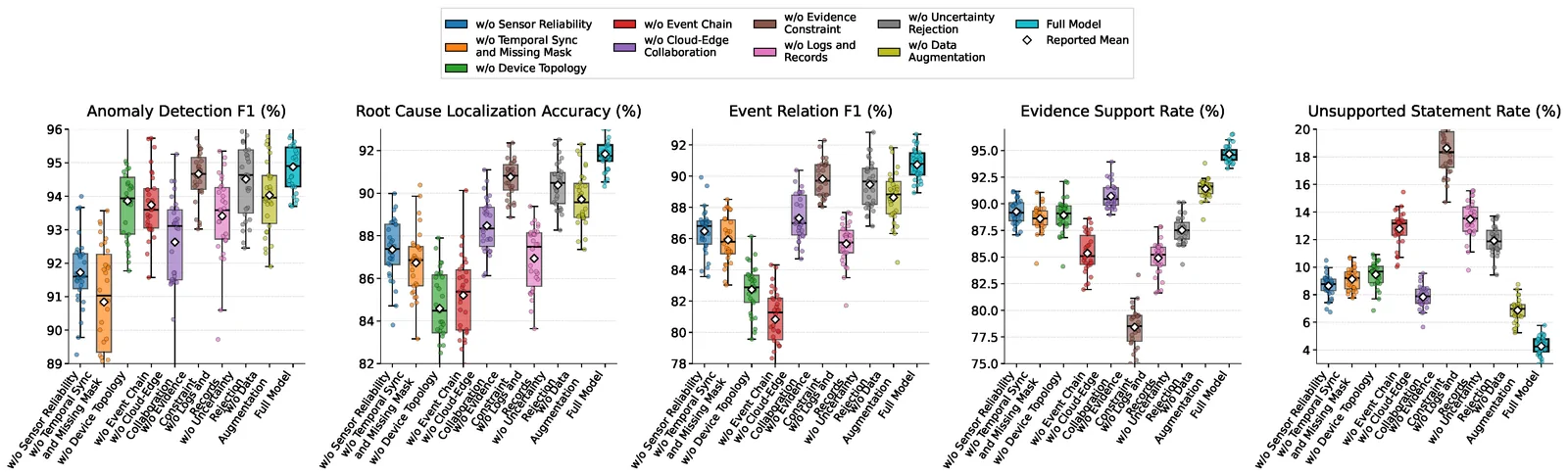
Distribution of ablation results across anomaly detection F1, root-cause localization accuracy, event relation F1, evidence support rate, and unsupported statement rate, with the full model achieving the best performance on all positive metrics and the lowest unsupported statement rate.

**Table 1:** Composition and scale of the banking data-center IT operational dataset.

Data Type	Sensor Type or Model	Data Volume
CPU operating-state data	Integrated digital temperature sensors and performance counters of Intel Xeon processors	1,286,400 records
GPU operating-state data	NVIDIA Data Center GPU Manager telemetry sensors	972,000 records
Power, current, and voltage data	Dell iDRAC 9 power telemetry module and intelligent PDU	1,036,800 records
Fan-speed and thermal-state data	Dell PowerEdge fan-speed sensors	518,400 records
Memory and disk operating data	Prometheus Node Exporter and S.M.A.R.T. sensors	864,000 records
Cabinet temperature and humidity data	Sensirion SHT31 temperature and humidity sensor	432,000 records
Network latency and packet-loss data	Cisco ISR 4331 network telemetry module	691,200 records
Switch port traffic data	Cisco Catalyst 9300 NetFlow/IPFIX sensors	1,036,800 records
Task-queue and service-load data	Prometheus application performance monitoring interfaces	604,800 records
System and application logs	Journald, Windows Event Log, Syslog, and ELK log collectors	187,420 records
Device alarm data	Dell OpenManage, Zabbix, and security operations platform alarm interfaces	16,382 records
Maintenance and fault tickets	IT service management system	5946 records
Configuration changes and operation records	CMDB, bastion host, and operation and maintenance audit platform	38,672 records
Device topology and service dependency relationships	LLDP/CDP, CMDB, and service call-chain collection interfaces	12,486 relationships
Laws, regulations, and regulatory standards	National Database of Laws and Regulations and public financial regulatory platforms	1268 documents
Judicial cases and administrative penalty decisions	Public judicial case platforms and regulatory penalty announcements	2416 documents

**Table 2:** Dataset allocation and expected training exposure under online augmentation.

Data Group	Original Total	Training Before Augmentation	Validation	Test	Training After Augmentation
Continuous sensor records	7,442,400	5,209,680	1,116,360	1,116,360	6,772,584 expected record equivalents
Aligned discrete events	248,420	173,894	37,263	37,263	not independently duplicated

**Table 3:** Performance comparison of different methods on the hardware anomaly detection task. Results are reported as the mean ± 95% confidence interval over multiple independent experiments.

Method	Accuracy (%) ↑	Precision (%) ↑	Recall (%) ↑	F1-Score (%) ↑	AUC (%) ↑
Random Forest	84.26±0.71	83.79±0.76	82.94±0.82	83.36±0.74	88.63±0.65
XGBoost	86.17±0.65	85.72±0.69	84.83±0.75	85.27±0.67	90.54±0.58
LSTM	87.84±0.61	87.31±0.64	86.92±0.69	87.11±0.62	91.76±0.54
TCN	89.43±0.56	89.06±0.58	88.47±0.63	88.76±0.57	93.18±0.49
Transformer	90.21±0.52	89.84±0.55	89.36±0.59	89.60±0.53	93.91±0.45
PatchTST	91.37±0.47	91.02±0.49	90.41±0.54	90.71±0.48	94.82±0.41
Early Fusion	89.76±0.54	89.28±0.57	88.93±0.61	89.10±0.55	93.46±0.47
Cross-modal Transformer	92.48±0.43	92.16±0.45	91.72±0.49	91.94±0.44	95.63±0.37
Graph Neural Network	$\underline{93.26\pm 0.39}$	$\underline{92.91\pm 0.41}$	$\underline{92.48\pm 0.45}$	$\underline{92.69\pm 0.40}$	$\underline{96.21\pm 0.34}$
Proposed Method	95.38±0.31	95.06±0.33	94.71±0.36	94.88±0.32	97.41±0.27

Bold and underlined values indicate the best and second-best mean results, respectively.

**Table 4:** Performance comparison of different methods on root-cause localization and anomaly propagation analysis tasks. Results are reported as the mean ± 95% confidence interval over multiple independent experiments.

Method	Root-Cause Localization Accuracy (%) ↑	Top-3 Accuracy (%) ↑	MRR ↑	Event Relation F1 (%) ↑	Temporal Localization Error (s) ↓
Random Forest	68.42±0.91	81.37±0.76	0.749±0.011	65.83±0.94	6.84±0.23
XGBoost	71.65±0.84	84.29±0.69	0.778±0.010	69.47±0.86	6.21±0.21
LSTM	74.38±0.78	86.42±0.64	0.801±0.009	72.16±0.81	5.48±0.19
TCN	77.91±0.71	88.74±0.58	0.829±0.008	75.83±0.74	4.76±0.17
Transformer	79.46±0.67	89.86±0.55	0.842±0.008	77.34±0.70	4.42±0.16
PatchTST	81.73±0.61	91.38±0.49	0.861±0.007	79.68±0.64	3.91±0.14
Early Fusion	78.84±0.69	89.27±0.56	0.836±0.008	76.59±0.72	4.58±0.16
Cross-modal Transformer	85.37±0.52	93.84±0.42	0.892±0.006	84.21±0.54	3.14±0.12
Graph Neural Network	$\underline{88.62\pm 0.44}$	$\underline{95.31\pm 0.36}$	$\underline{0.918\pm 0.005}$	$\underline{87.46\pm 0.47}$	$\underline{2.47\pm 0.10}$
Proposed Method	91.84±0.36	97.26±0.28	0.944±0.004	90.73±0.39	1.82±0.08

Bold and underlined values indicate the best and second-best mean results, respectively.

**Table 5:** Ablation study results under different model configurations. Results are reported as the mean ± 95% confidence interval over multiple independent experiments.

Model Configuration	Anomaly Detection F1 (%) ↑	Root-Cause Localization Accuracy (%) ↑	Event Relation F1 (%) ↑	Evidence Support Rate (%) ↑	Unsupported Statement Rate (%) ↓
w/o Sensor Reliability Estimation	91.72±0.45	87.36±0.49	86.48±0.51	89.27±0.44	8.64±0.31
w/o Temporal Synchronization and Missing Mask	90.84±0.49	86.73±0.53	85.92±0.55	88.61±0.47	9.12±0.34
w/o Device Topology Modeling	93.86±0.37	84.59±0.57	82.74±0.61	88.93±0.46	9.46±0.35
w/o Anomaly Event Chain Module	93.74±0.38	85.21±0.55	80.83±0.66	85.36±0.53	12.78±0.42
w/o Cloud–Edge Dynamic Collaboration	92.63±0.42	88.47±0.47	87.31±0.49	90.72±0.41	7.83±0.29
RAG-only LLM (w/o Evidence Gate, Mask, and Rejection)	94.67±0.33	90.76±0.39	89.82±0.42	78.42±0.68	18.63±0.57
w/o Logs and Operation Records	93.41±0.39	86.94±0.51	85.67±0.54	84.91±0.56	13.47±0.45
w/o Uncertainty Rejection Mechanism	94.51±0.34	90.38±0.40	89.46±0.43	87.53±0.49	11.92±0.39
w/o Robust Data Augmentation	$\underline{94.03\pm 0.36}$	$\underline{89.71\pm 0.43}$	$\underline{88.64\pm 0.46}$	$\underline{91.42\pm 0.39}$	$\underline{6.87\pm 0.26}$
Full Model	94.88±0.32	91.84±0.36	90.73±0.39	94.68±0.32	4.26±0.21

Bold values indicate the best mean results; underlining identifies the configuration without robust data augmentation.

## Data Availability

The data presented in this study are available on request from the corresponding author.
